# Anti-Inflammatory Properties of Statin-Loaded Biodegradable Lecithin/Chitosan Nanoparticles: A Step Toward Nose-to-Brain Treatment of Neurodegenerative Diseases

**DOI:** 10.3389/fphar.2021.716380

**Published:** 2021-09-24

**Authors:** Adryana Rocha Clementino, Cinzia Marchi, Michele Pozzoli, Franco Bernini, Francesca Zimetti, Fabio Sonvico

**Affiliations:** ^1^ Department of Food and Drug, University of Parma, Parma, Italy; ^2^ Conselho Nacional do Desenvolvimento Científico e Tecnológico-CNPq, Brasilia, Brazil; ^3^ The Woolcock Institute for Medical Research, Discipline of Pharmacology, Sydney Medical School, University of Sydney, Sydney, NSW, Australia; ^4^ University Research Centre for the Innovation of Health Products (Biopharmanet-TEC), University of Parma, Parma, Italy

**Keywords:** hybrid nanoparticles, enzymatic degradation, RPMI2650 cell line, statins, neuroinflammation, microglia-like THP-1 cell line, nasal drug delivery

## Abstract

Nasal delivery has been indicated as one of the most interesting alternative routes for the brain delivery of neuroprotective drugs. Nanocarriers have emerged as a promising strategy for the delivery of neurotherapeutics across the nasal epithelia. In this work, hybrid lecithin/chitosan nanoparticles (LCNs) were proposed as a drug delivery platform for the nasal administration of simvastatin (SVT) for the treatment of neuroinflammatory diseases. The impact of SVT nanoencapsulation on its transport across the nasal epithelium was investigated, as well as the efficacy of SVT-LCNs in suppressing cytokines release in a cellular model of neuroinflammation. Drug release studies were performed in simulated nasal fluids to investigate SVT release from the nanoparticles under conditions mimicking the physiological environment present in the nasal cavity. It was observed that interaction of nanoparticles with a simulated nasal mucus decreased nanoparticle drug release and/or slowed drug diffusion. On the other hand, it was demonstrated that two antibacterial enzymes commonly present in the nasal secretions, lysozyme and phospholipase A2, promoted drug release from the nanocarrier. Indeed, an enzyme-triggered drug release was observed even in the presence of mucus, with a 5-fold increase in drug release from LCNs. Moreover, chitosan-coated nanoparticles enhanced SVT permeation across a human cell model of the nasal epithelium (×11). The nanoformulation pharmacological activity was assessed using an accepted model of microglia, obtained by activating the human macrophage cell line THP-1 with the *Escherichia coli*–derived lipopolysaccharide (LPS) as the pro-inflammatory stimulus. SVT-LCNs were demonstrated to suppress the pro-inflammatory signaling more efficiently than the simple drug solution (−75% for IL-6 and −27% for TNF-α vs*.* −47% and −15% at 10 µM concentration for SVT-LCNs and SVT solution, respectively). Moreover, neither cellular toxicity nor pro-inflammatory responses were evidenced for the treatment with the blank nanoparticles even after 36 h of incubation, indicating a good biocompatibility of the nanomedicine components *in vitro*. Due to their biocompatibility and ability to promote drug release and absorption at the biointerface, hybrid LCNs appear to be an ideal carrier for achieving nose-to-brain delivery of poorly water-soluble drugs such as SVT.

## Introduction

The term “neuroinflammation” is used to describe the inflammatory response originated in the central nervous system (CNS) after local damage signals, including trauma, infectious agents, oxidative species release, β-amyloid oligomers, and *τ* protein neurofibrillary tangle formation (Morales et al., 2014). Immune responses in the CNS occur very frequently and are mostly mediated by resident innate glial immune cells. Indeed, microglia represent the major cellular component of the innate immune system of the brain. An acute inflammatory response in the CNS is caused by the immediate and early activation of microglia cells in response to noxious stimuli and by the expression of pro-inflammatory mediators, such as cytokines and reactive oxygen species (ROS) (Fakhoury, 2016). The formation and release of protective/resolving inflammatory mediators is a defensive response contribution to tissue repair and resolution of inflammation. However, if the stimulus remains persistent, a pathological chronic inflammatory condition can develop, causing cumulative damage over time. The hyperactivation of microglia cells can cause an excessive release of cytotoxic factors, with consequent deleterious effects also for healthy tissues, especially on the neuronal cells. Indeed, recent data suggest that local brain immune activation and evolution of the inflammation response have the capacity to facilitate and trigger the pathophysiological conditions typical for many neurodegenerative diseases (Hennessy et al., 2015). Apparently, glial cells secrete and respond to a vast array of signaling molecules, producing exaggerated amounts of chemokines (CCL2 and CXCL1) and cytokines (IL-6, TNF-α, and IL-1β) (Elain et al., 2014). Elevated levels of chemo/cytokines have been detected in the serum and cerebrospinal fluids of patients suffering from Alzheimer’s disease (AD), Parkinson’s disease (PD), multiple sclerosis (MS), and Huntington’s disease (HD). Together with other factors, these elevated chemo/cytokine levels appear to play an important role in the neurodegenerative processes of these illnesses since these diseases share the ubiquitous feature of chronic inflammation (Erta et al., 2012). In AD and depression animal models, the release of neuroinflammatory cytokines appears to have a role in the pathogenesis mediated by increased levels of β-amyloid, either *via* reduced clearance or increased production from the amyloid precursor protein (Alasmari et al., 2018; Morgese et al., 2020). Moreover, mounting evidence indicates that the oxidative and inflammatory imbalance accompanying cardiovascular disease could be related to the pathogenesis and progression of AD. In particular, the alteration of the proteic composition of high-density lipoproteins (HDLs) and the reduction of their antioxidant and protective properties on neurovasculature appear relevant for AD development (Zimetti et al., 2021).

The need for effective treatments to prevent neuroinflammation-related damage associated with neurodegenerative diseases has boosted the interest in drug repurposing (Athauda and Foltynie, 2018; Ballard et al., 2020). In fact, it is believed that existing drugs with multiple pharmacological effects against the neuroinflammation genesis and progression could contribute to address the multifaceted nature of neurodegenerative pathology and/or diminish the cumulative effects of inflammation in the brain. In this regard, in the past few years, our group has proposed, for the first time, the nose-to-brain delivery of encapsulated statins as an innovative approach in the treatment of neurodegenerative diseases (Clementino et al., 2016). Statins, as a result of HMG-CoA reductase inhibition, are lipid-lowering agents widely used as a first-line therapy in cholesterol-related cardiovascular diseases.

However, several clinical investigations have also suggested a beneficial role of statins on the pathogenesis of AD (Sodero and Barrantes, 2020) *via* the modulation of brain cholesterol levels as well as through pleiotropic effects, acting, for example, on β-amyloid production and accumulation in plaques (Fassbender et al., 2001; Ehehalt et al., 2003). Clinical–mechanistic studies evidenced that simvastatin (SVT) is able to modulate the microglia activity and the release of inflammatory markers in the injured brain (Li et al., 2009; Manickavasagam et al., 2020). Furthermore, statins can suppress cellular apoptosis in the CNS, stimulate the expression of neurotrophic factors (NGF and BDNFG) (Yang et al., 2012), and reduce the expression of inducible nitric oxide synthase (iNOS) (Roy and Pahan, 2011) while maintaining the blood–brain barrier (BBB) integrity. However, these effects have proven to be quite elusive after oral administration, possibly as a direct consequence of high hepatic extraction and poor crossing of the BBB (McGuinness et al., 2014; Sonvico et al., 2017). Importantly, an efficient delivery of statins to the cerebral parenchyma could be decisive for the management of several neurodegenerative disorders.

Recently, nasal drug administration has been indicated as one of the most interesting alternative routes for brain delivery. In fact, nasal delivery allows the avoidance of systemic drug metabolism and the necessity of drug transport across the BBB (Illum, 2000). In addition, intranasal administration, being noninvasive and not requiring the intervention of healthcare professionals, may offer additional advantages in terms of patient compliance and enhanced adherence to the therapy in the case of chronic therapy. However, the extent of the nasal drug absorption is highly dependent on the formulation and on its residence time on the nasal mucosa, even more so in the case of nose-to-brain delivery (Wu et al., 2008). Consequently, the ability to deliver therapeutically relevant amounts of drugs directly from the nose to brain strictly depends on the availability of efficient drug delivery systems (Mistry et al., 2015).

Lecithin/chitosan nanoparticles (LCNs) have been proposed as a delivery system for hydrophobic drugs through mucosal tissues (Barbieri et al., 2015; Şenyiğit et al., 2016). The chitosan surface layer characterizing their structure appears adapted for nasal delivery as it promotes mucoadhesion and enhances epithelial permeability (Gerelli et al., 2010; Bruinsmann et al., 2019). However, the idea that slow drug–releasing nanoparticles would provide a significant improvement of nose-to-brain delivery has been questioned (Illum, 2007). Conversely, rapidly biodegraded nanoparticles could improve the brain target and bioavailability of lipophilic drugs, by tailoring prompt drug release and absorption across the nasal mucosal surface. Previously, we developed simvastatin-loaded lecithin/chitosan nanoparticles (SVT-LCNs) presenting several desirable features in view of nose-to-brain delivery (Clementino et al., 2016). Small particle size, narrow size distribution, high positive surface charge, and high drug-encapsulation efficiency and stability were obtained for nanoparticles produced with an efficient self-assembly process. Hence, in the present work, we carefully investigated features of the nanoformulation that are critical for SVT to enter the cerebral parenchyma while exploiting the nasal route of administration. In particular, nanoparticles’ composition, physicochemical properties affecting drug release, interaction of nanoparticles with mucosal surfaces, and drug transport across nasal epithelia were explored. Last, the efficacy of SVT-loaded hybrid LCNs in attenuating cytokine release was evaluated for the first time using a myeloid cell line as a model for microglia.

## Materials and Methods

### Materials

Chitosan (Chitoclear FG, deacetylation degree 95%, viscosity 45 cP) was provided by Primex (Siglufjordur, Iceland) and used without further purification. Lecithin (Lipoid S45) was obtained from Lipoid AG (Ludwigshafen, Germany). Pharmaceutical grade oils such as Maisine™ 35-1 (glycerol monolinoleate) and Labrafac™ Lipophile WL 1349 (medium chain triglycerides, EP) were a gift from Gattefossé (Saint-Priest, France). Simvastatin USP 99%, mucin from a porcine stomach (Type III), human lysozyme, and phospholipase A2 were supplied by Sigma–Aldrich (Steinheim, Germany).

Cell line RPMI 2650 (CCL-30) was purchased from American Type Culture Collection (ATCC, Manassas, VA, United States). Human monocyte THP-1 cell line was purchased from the European Collection of Authenticated Cell Culture (ECACC, Porton Down, United Kingdom). Minimum essential cell culture medium (MEM), RPMI 1640, and fetal bovine serum (FBS) were acquired from Life Technologies (Thermo Fisher Scientific, Monza, Italy). Cell culture inserts and other culture plastics were acquired from Corning^®^ Life Science (Tewksbury, MA, United States). Phorbol 12-myristate 13-acetate (PMA), lipopolysaccharide from *Escherichia coli*, and thiazolyl blue tetrazolium bromide (MTT) were obtained from Sigma–Aldrich (St. Louis, MO, United States). All other chemicals were of analytical grade. Ultrapure and degassed ultrapure water (Purelab Flex, ELGA-Veolia LabWater, High Wycombe, United Kingdom) were used in all experiments.

### Methods

#### Simvastatin-Loaded Lecithin/Chitosan Nanoparticle (SVT-LCN) Preparation

SVT-LCNs were prepared through a self-emulsifying process involving lecithin, a negatively charged phospholipid, and chitosan, a derivative of the natural polysaccharide chitin, according to a protocol previously reported (Clementino et al., 2016). Briefly, 10 mg of SVT was dissolved in 0.8 ml of an ethanol solution of lecithin (2.5%), containing 20 mg of both Labrafac and Maisine oil vehicles. Then, the lecithin solution containing SVT and oils was injected into 10 ml of a 0.01% w/v chitosan aqueous solution, under constant agitation (magnetic stirring, 300 rpm) and temperature (40°C). Freshly prepared nanoparticle suspensions were stirred at constant temperature until complete evaporation of ethanol from the system. SVT-LCNs were characterized for the particle size, polydispersity index, and surface zeta potential using a Malvern Zetasizer Nano ZSP (Malvern Instruments Ltd., United Kingdom). Nanoparticles’ encapsulation efficiency (EE) was determined indirectly by centrifugation and ultrafiltration (Vivaspin^®^ 2, MWCO 30,000, Sartorius, Germany) to separate nanoparticles from a nonencapsulated drug material, as previously reported (Bruinsmann et al., 2019). SVT content was quantified applying a validated high-performance liquid chromatography (HPLC) analytical method with a UV/Vis detector set at 238 nm and published elsewhere (Clementino and Sonvico, 2018).

#### Nanoparticle *In Vitro* Drug Release

*In vitro* drug release experiments were conducted using vertical Franz type diffusion cells (0.6 cm^2^ diffusional area) (Disa, Milan, Italy) assembled with a dialysis cellulose membrane (MWCO 14,000 Da, Sigma–Aldrich, St. Louis, MO, United States) separating the donor and receptor compartments. Receptor compartments were accurately filled with 4 ml of a simulated nasal electrolyte solution (SNES, pH 6.5), which is used as a release medium (Castile et al., 2013). Assembled Franz cells were maintained at constant temperature (37.0 ± 0.2°C) and magnetically stirred (800 rpm) during 24 h of the experiment. SVT-LCN drug release was performed dispersing 25 µl of formulation in 1) SNES, 2) an artificial mucus model (1% of porcine stomach mucin type II dispersed in SNES) (Griffiths et al., 2010), 3) SNES, or 4) artificial mucus containing 0.5 mg/ml of lysozyme and 0.6 µg/ml of phospholipase A2 (Sigma–Aldrich, St. Louis, MO, United States). Samples were then added to the donor compartment. At predetermined time points, aliquots of 500 µl were withdrawn from the receptor compartment and replaced with the same amount of fresh SNES medium to maintain *sink* conditions. Collected samples were analyzed by HPLC-UV for SVT content. At the end of the experiment, the content donor compartments from each Franz cell and the dialysis membranes were also assayed for SVT content to calculate the overall mass balance. SVT release experiments were performed at least in triplicate.

#### Study of SVT-LCN Permeation Across RPMI2650 Nasal Cells

*In vitro* permeation studies were performed against a cellular model of nasal mucosa, as previously reported (Pozzoli et al., 2016). Briefly, RPMI 2650 human nasal epithelial cell lines (ATCC, Manassas, VA, United States) were cultivated in the minimum essential medium (MEM, Life Technologies, Thermo Fisher Scientific, Monza, Italy) enriched with 10% (v/v) of fetal bovine serum (FBS) and 1% (v/v) of nonessential amino acid solution, and incubated at 37°C with 95% air humidity and 5% CO_2_ atmosphere. When confluence was 90%, cells were seeded at the concentration of 2.5 × 10^6^ cells/ml on collagen-coated Snapwell^®^ polyester membrane inserts (Costar, Corning Life Sciences, Tewksbury, MA, United States, 1.13 cm^2^, 0.4 µm pore size, coated with 200 µl of 1 × collagen PBS solution 24 h prior to seeding). Then, 24 h after seeding, the medium on the apical chamber was completely removed to transfer cells to an air–liquid interface (ALI) condition, allowing cells to differentiate in a pseudostratified monolayer. Acceptor compartments were filled with 1.5 ml of the cell culture medium and replaced 3 times *per* week. Transepithelial electric resistance (TEER) measurements were recorded using an EVOM2 epithelial voltohmmeter (World Precision Instruments Inc., Sarosata, FL, United States) to evaluate the monolayer integrity and suitability for the transport studies. Finally, 14 days after seeding, 250 µl of 1 mg/ml SVT-LCNs or 1 mg/ml simvastatin suspension in HBSS, which was used as a control, was added on the cell surface to carry out permeation studies, in triplicate. Samples of 200 µl were collected from the acceptor compartment every hour and analyzed by HPLC-UV for SVT content. After 4 h, the apical surface of cell epithelia was washed twice with HBSS to collect any remaining non-permeated drug. Thereafter, cells were scraped out from SnapWell^®^ inserts and lysed with CelLytic™ buffer (Invitrogen, Thermo Fisher Scientific, Waltham, MA, United States) to extract and quantify the amount of SVT accumulated within the cells.

#### Effect of SVT-LCNs on Pro-Inflammatory Glia-Like Cell Viability

To evaluate the impact of SVT-LCNs on cell viability, human monocyte-derived macrophages THP-1 were used. These cells are widely accepted as the surrogate of microglial cells to evaluate cytokine secretion in the context of neuroinflammation in AD (McFarland et al., 2017; Wiatrak and Balon, 2021). THP-1 monocytes were cultured in RPMI 1640 medium supplemented with 10% FCS at 37°C in 5% CO_2_. To perform the experiments, cells were seeded in 24-well plates at the density of 5·10^5^ cells/well in the presence of 100 ng/ml PMA for 72 h to allow differentiation into macrophages. After differentiation, cells were treated for 2 h with lipopolysaccharide from *E. coli* (LPS, 0.25 μg/ml) as pro-inflammatory stimulus (Wiatrak and Balon, 2021). Cells were then treated with SVT-LCNs or with SVT dissolved in DMSO at concentrations of 0.1, 1, and 10 µM. After 36 h of incubation, the MTT assay was performed on cell monolayers. In detail, SVT-LCN and SVT treatments were stopped, and cells were treated with a solution of 1 mg/ml of MTT in RPMI supplemented with 5% FBS, at 37°C for 2 h in the dark. The solution was then removed, and the resulting intracellular formazan crystals were dissolved in 200 µl of DMSO under shaking for 10 min. Finally, absorbance was measured at a wavelength of 570 nm using an absorbance microplate reader (Spark^®^, Tecan, Männedorf, Switzerland). Absorbance values were directly correlated with the cellular viability, and the percentage of cell viability for each treatment was compared to control values obtained for untreated cells.

#### Investigation of the Anti-Inflammatory Activity of SVT-LCNs

In order to assess the anti-inflammatory activity of SVT-LCNs, THP-1 cells were seeded and differentiated as previously described, treated for 2 h with LPS, and subsequently incubated with fresh RPMI for 6 h in order to promote cytokine secretion and accumulation in the medium. Initially, an evaluation at different incubation times for both SVT-LCNs and SVT solution was performed to investigate the optimal treatment conditions. In the first condition tested (indicated as protocol A), SVT or SVT-LCNs at 10 µM were added 24 h before and during the 2 h treatment with LPS. In the second condition (indicated as protocol B), cells were treated with free SVT or SVT-LCNs at 10 µM before and during incubation with LPS, as well as for further 6 h after the treatment with LPS. Once the optimal experimental condition was identified, which was set as protocol B, THP-1 cells were finally treated with increasing drug concentrations of SVT-LCNs and SVT (0.1, 1, and 10 μM), for comparison purposes. At the end of the treatment, supernatants were collected, and IL-6 and TNF-α production in the medium was quantified by ELISA assay kits according to the manufacturer's instructions (Thermo Fischer Scientific, Waltham, MA, United States).

#### Statistics

The statistical analyses were performed with Prism 8 software (GraphPad Software, San Diego, CA, United States). Each experiment was run at least in triplicate, and results were expressed with the mean and the standard deviation. The results were analyzed using Student’s *t*-test for unpaired samples when comparing two groups (indicated as T in Figure legends) or ordinary one-way ANOVA to compare more than two conditions, applying the Tukey *post hoc* test for multiple comparisons (indicated as F in Figure legends).

## Results

### Physicochemical Characterization of SVT-LCNs

SVT-LCNs were formed by the electrostatic self-assembly of lecithin and chitosan and have a multilayered structure alternating chitosan aqueous-rich layers and phospholipid bilayers, as previously reported (Gerelli et al., 2008b; Gerelli et al., 2008a). In order to improve drug encapsulation efficiency, nanoparticles were produced with the addition of pharmaceutical grade oils to help drug encapsulation in the lipophilic domain of LCNs. The physicochemical characteristics of SVT-LCNs are presented in Table 1. The encapsulation of the statin into the LCN structure did not affect the main physicochemical features of the nanomedicine. SVT-LCNs showed a relatively small particle size (218 ± 12 nm), with narrow size distribution (PDI 0.096 ± 0.032) and positive surface charge (+44.3 ± 2.1 mV). High drug encapsulation efficiency was achieved with nanoparticles encapsulating 98% (±1.2%) of the total amount of SVT used in the preparation (1 mg/ml).

**TABLE 1:** Nanoparticles’ physicochemical properties and simvastatin encapsulation efficiency (EE).

Batch code	Particle size (nm)	ζ Potential (mV)	PDI	EE (%)
Blank LCN	147.0 ± 26.4	+57.3 ± 2.6	0.380 ± 0.025	—
SVT-LCN	217.8 ± 12.1	+44.3 ± 2.1	0.096 ± 0.032	98.0 ± 1.2

### SVT Release From SVT-LCNs in Biorelevant Conditions

**FIGURE 1:**
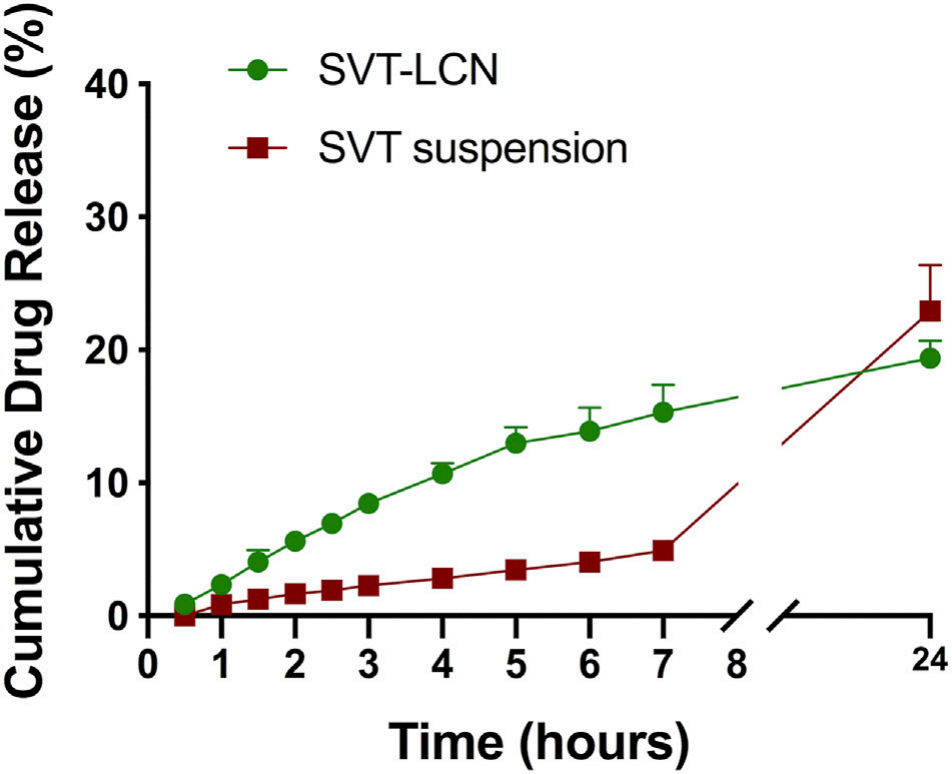
Simvastatin release from drug suspension (red squares) and SVT-LCNs (green dots) in simulated nasal electrolyte solution (SNES), pH 6.5 (*n* = 3, ± S.D.). Experiments were carried out in a vertical Franz cell apparatus using a cellulose dialysis membrane (MWCO 14,000 Da).

To investigate the hybrid nanoparticle drug release *in vitro*, a simple electrolyte solution containing sodium, potassium, and calcium salts (SNES, pH 6.5) was initially used as the release medium to simulate nasal secretions. Release studies were performed using vertical Franz diffusion cells and a semipermeable membrane to separate released/dissolved drug from drug-releasing nanoparticles. Drug release profiles obtained for SVT-LCNs and SVT suspension in SNES are presented in Figure 1. Although no significant difference was found between the amount of SVT released from nanoparticles and from the suspension (approximately 20% of the drug was released) during the 24 h of the release experiment, nonencapsulated SVT showed an extremely low dissolution rate at early time points while the release from nanoparticles was prompt. In fact, over the first 7 h of the experiment, 15.31% (±1.06%) of SVT was released from the nanoparticles, displaying a significantly faster release in comparison to the SVT suspension (4.93 ± 0.57%).

**FIGURE 2:**
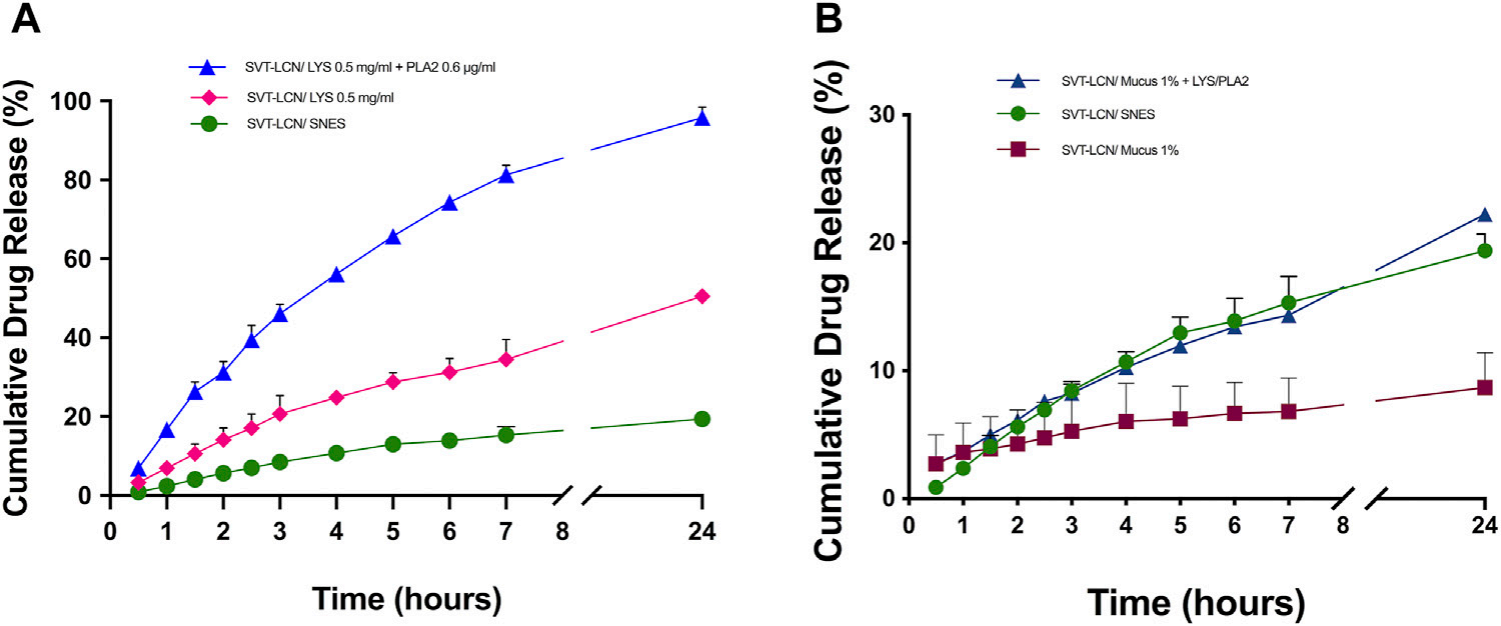
Panel **(A)-**SVT-LCN release in simulated nasal electrolyte solution (SNES) pH 6.5 (green dots) and with the addition of lysozyme 0.5 mg/ml (pink diamonds) and lysozyme 0.5 mg/ml + phospholipase A2 (PLA2) 0.6 µg/ml (blue triangles). Panel **(B)-**SVT-LCN release in SNES (green dots), simulated nasal mucus (SNES plus porcine stomach mucin type II at 1% w/v, pH 6.5; red squares) and with the addition of lysozyme 0.5 mg/ml + phospholipase A2 (PLA2) 0.6 µg/ml (blue triangles). Experiments (*n* = 3, ± S.D.) were carried out in a vertical Franz cell apparatus using a cellulose dialysis membrane over a 24-h period.

Subsequently, with the aim to simulate the physiological conditions present in the nasal cavity more closely, drug release studies were carried out using SNES containing mucus and/or two antibacterial enzymes physiologically present in nasal secretions, that is, lysozyme and phospholipase A2 (PLA2) as dissolution media (Travis et al., 2001). While, as shown previously, in the simple SNES electrolyte solution, SVT was released from SVT-LCNs in a controlled manner, a rather different behavior was observed when enzymes were added to SNES (Figure 2A). When lysozyme was added to SNES, the total drug release from SVT-LCNs doubled, attaining 50% at 24 h. Interestingly, when both lysozyme and PLA2 were added to the dissolution medium, nearly all the encapsulated SVT was released (96.82 ± 2.62%) within 24 h. Finally, to understand how mucus could impact nanoparticle delivery following nasal administration, *in vitro* release studies were performed using a simulated nasal mucus. Figure 2B illustrates that when nanoparticles were dispersed in the artificial mucus (SNES containing 1% porcine mucin), SVT release was significantly hindered (19.40 ± 1.30% vs. 8.69 ± 2.63%). However, when the enzymes were added to the simulated nasal mucus, the total release of SVT was boosted back up to 22%.

### SVT Permeation Across an *In Vitro* Model of the Nasal Epithelium

**FIGURE 3:**
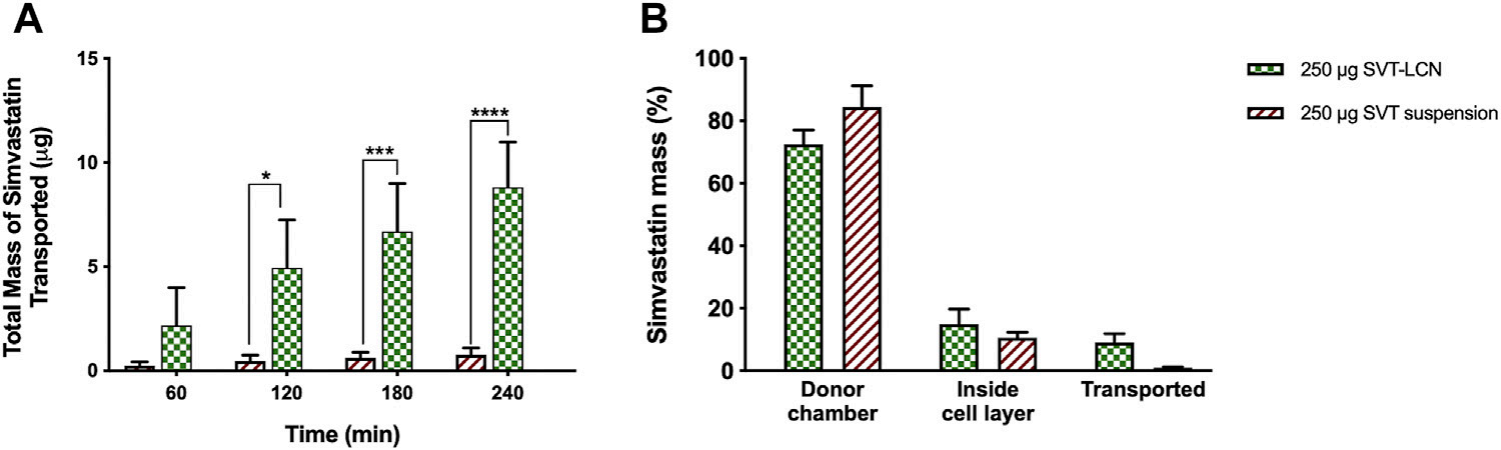
Panel **(A)**–Amount of simvastatin transported across the RPMI2650 nasal cell pseudo-monolayer over time after deposition on the cells apical surface of simvastatin-suspension (red bars) or simvastatin-loaded nanoparticles (SVT-LCNs, green bars). Panel **(B)**–Percentage distribution of total simvastatin recovered after 4 h of transport studies across the RPMI2650 nasal cell pseudo-monolayer using simvastatin suspension (red bars) or simvastatin-loaded nanoparticles (SVT-LCNs, green bars) (*n* = 3, ± S.D.). Permeability experiments were carried out on RPMI2650 cells grown under air–liquid interface conditions (TEER values were around 120 Ω·cm^2^ before and after transport studies).

The ability of SVT-LCNs to promote SVT absorption through the nasal mucosa was investigated using a mucus-producing the nasal epithelium model obtained using the human nasal septum cell line RPMI 2650 (Pozzoli et al., 2017). Figure 3A illustrates the total amount of SVT which had permeated across the nasal epithelial model after the deposition of SVT suspension and SVT-LCNs on the cells' pseudo-monolayer surface. After the first hour, approximately 3 µg (±1.8 µg) of SVT from SVT-LCNs was already transported, while only 0.25 µg (±0.17 µg) of SVT was found in the acceptor compartment of the drug suspension used as a control. At the end of the experiment after 4 h, 9 µg (±2.16 µg) of SVT from nanoparticles permeated the nasal cells vs just 0.8 µg (±0.34 µg) for SVT suspension (11-fold increase; *p* < 0.001). It was important to note that a progressive, almost linear, increase in the drug permeation for simvastatin-loaded nanoparticles was evidenced, while the permeation from simvastatin suspension occurred at a very slow rate, reaching almost a plateau. These data suggest an enhanced drug permeability when lecithin/chitosan nanoparticle technology is applied.

As shown in Figure 3B, after 4 h of permeation, 84.42 ± 6.79% and 72.94 ± 4.52% of the drug remained on the surface of the cell layer for SVT suspension and SVT-LCNs, respectively. Around 14.94 ± 4.75% of SVT from the nanoparticle formulation and 11.03 ± 1.74% from the SVT suspension were found inside the cells, suggesting a higher internalization of nanoparticles within the cells of the nasal epithelium.

### Investigation of SVT-LCN Cytotoxicity Against the Glia-Like THP-1 Human Cell Line

The biocompatibility of the nanoparticle formulation on the THP-1 cell line was assessed by determining cell viability followed by 36 h incubation and using SVT solution in DMSO as control. Results are reported in Supplementary Materials (Supplementary Figure 1) and show that neither the raw material (SVT) nor the nanoparticle formulation (SVT-LCNs) affected the glia-like cells' viability at the drug concentrations tested (0.1, 1, and 10 µM). Indeed, cell viability remained around 100% for all treated groups compared to untreated control cells.

### Investigation of the Anti-Inflammatory Activity of SVT-LCNs

The *in vitro* efficacy of SVT-LCN formulation was further assessed using LPS-treated glia-like THP-1 as a surrogate model of neuroinflammation (McFarland et al., 2017). Time- and dose-dependent effects of LPS stimulation on TNF-α and IL-6 release from THP-1 cells were evaluated and are presented in the Supplementary Material. Experimental evidence showed that LPS has a significant pro-inflammatory effect in the glia-like cell model at the concentration of 0.25 µg/ml with a sustained release of TNF-α and IL-6 pro-inflammatory cytokines observed beyond the 2 h of incubation with LPS (data not shown) and attained a peak concentration after 6 h (Supplementary Figure 2).

**FIGURE 4:**
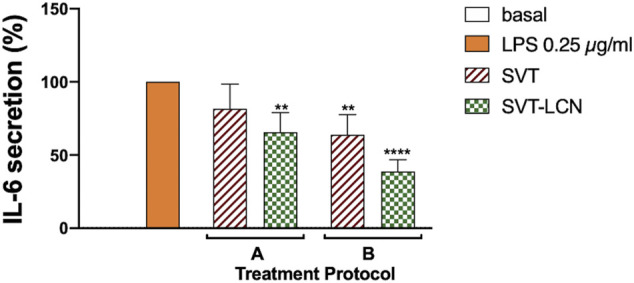
Effect of treatment with 10 μM SVT solution and SVT-LCNs on the secretion of IL-6 from THP-1 glia-like cells according to two different treatment protocols: A-cells were pretreated with 10 µM of SVT solution or SVT-LCNs for 24 h followed by 2 h of simultaneous treatment with LPS 0.25 µg/ml; B-cells were treated with 10 µM of SVT solution or SVT-LCNs prior (24 h), during (2 h), and after LPS treatment (6 h). Data are expressed as mean percentage ± S.D. of cytokine secretion from at least three repeated measurements. Statistical significance of the results is expressed with respect to the LPS control treatment (*: *p* < 0.05; **p < 0.01 compared to LPS, F).

The release of pro-inflammatory cytokines followed by LPS stimuli was used to evaluate the potential effect of SVT-LCNs to counteract neuroinflammation. The experimental conditions were designed and optimized to simulate a preventive and a chronic neuroinflammation treatment. LPS-treated cells were exposed to two different treatment protocols with SVT solution or SVT-LCNs (Figure 4). In the first case, protocol A, cells were pretreated with 10 µM of SVT solution or SVT-LCNs for 24 h, followed by 2 h of simultaneous treatment with 0.25 µg/ml of LPS (total of 26 h incubation). Conversely, for protocol B, cells were treated with 10 µM of SVT solution or SVT-LCNs prior (24 h), during (2 h), and after (6 h) the LPS stimulus (total of 32 h of incubation).

**FIGURE 5:**
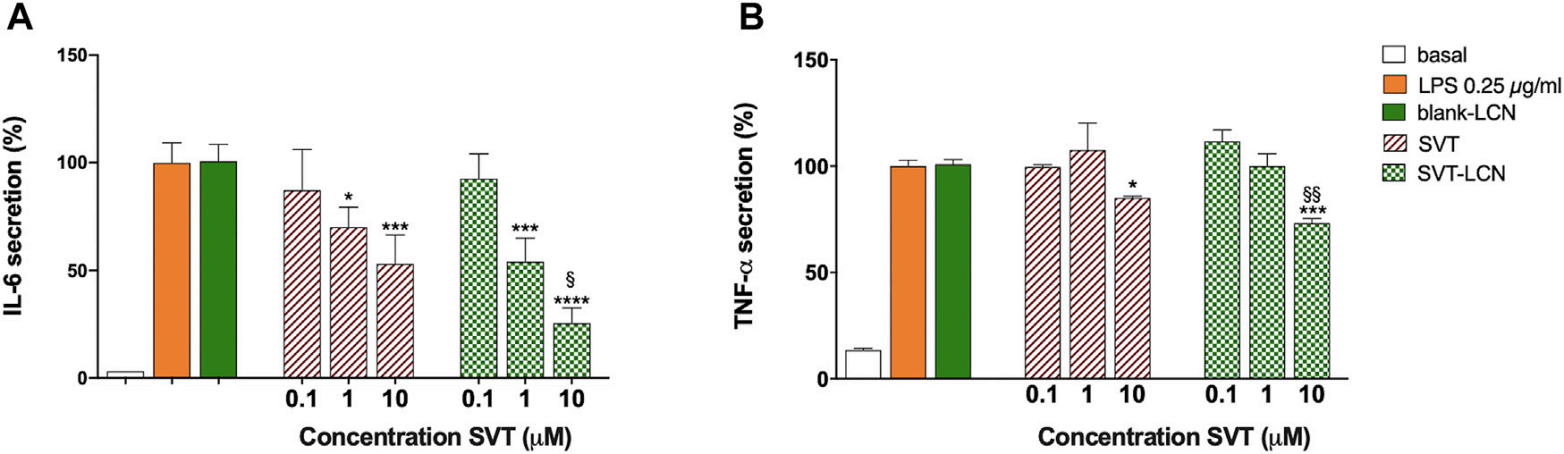
Dose–response evaluation of the treatment with SVT solution and SVT-LCNs on the secretion of IL-6 **(A)** and TNF-a **(B)** from THP-1 glia-like cells after LPS treatment (0.25 µg/ml) following protocol B as shown in Figure 4. Basal cytokine levels, LPS stimulus alone, and blank LCNs (the nanoparticle amount corresponding to 10 µM of SVT) are presented as controls. Data are expressed as mean percentage ± S.D. of cytokine secretion from at least three repeated measurements. Statistical significance of the results is expressed in comparison to that of the LPS treatment (*p < 0.05; ***p < 0.001; p****<0.0001) or to 10 µM SVT solution (§ *p* < 0.05; §§ *p* < 0.01, T).

For both protocols and treatments, that is, SVT solution and SVT-LCNs, a reduction in LPS-induced IL-6 cytokine release from the THP-1 cells was observed. However, considering the first treatment condition (Figure 4, A protocol), only SVT-LCNs displayed a statistically significant reduction of IL-6 levels (−37% IL-6 concentration vs. LPS, *p* < 0.05). Nevertheless, a significant reduction in LPS-induced IL-6 secretion was observed, for both SVT solution (−38% IL-6 concentration vs. LPS, *p* < 0.05) and SVT-LCNs (−63% IL-6 concentration vs. LPS, *p* < 0.01), when the treatments were simulating a chronic treatment (Figure 4, Protocol B). Then, the extent to which SVT-LCN treatment would impact the pro-inflammatory signaling and microglia activation was assessed by measuring IL-6 and TNF-α release by THP-1 glia-like cells as a consequence of the LPS stimulus. The SVT solution was used as the positive control for drug efficacy, while blank nanoparticles were tested to exclude any effect by the nanocarrier components. SVT-LCNs and the SVT solution exhibited a dose-dependent reduction of the activation of glia-like THP-1 cells in terms of release of IL-6 (Figure 5A). In the case of TNF-α, only the highest investigated drug concentration was able to significantly reduce the cytokine secretion for both treatments (Figure 5B). However, only in the case of treatment with SVT-LCNs, the attenuation of cytokine release has been relevant. In fact following LPS stimulation, only the treatment with SVT-LCNs (10 µM) showed at the same time a highly significant suppression of IL-6 release (−75% IL-6 concentration vs*.* LPS, *p* < 0.001) and of TNF-α levels (−27% of TNF-α levels vs*.* LPS, *p* < 0.01). Moreover, the reduction induced by SVT-LCNs was more pronounced than that of SVT solution (*p* = 0.05 and *p* = 0.005 for IL-6 and TNF-α, respectively). Additionally, blank LCNs did not show pharmacological activity, with cytokine levels superimposable to those obtained for the LPS stimulus alone, ruling out a potential anti-inflammatory activity from the nanoparticle components.

## Discussion

The nose is an attractive alternative route for delivery of drugs to the brain. However, the complexity of the nasal anatomy and physiology can pose a remarkable challenge in achieving the delivery of intranasally administered drugs to the brain. The use of nanomedicine is a promising approach for the nose-to-brain transport of therapeutics across the nasal mucosa (Mistry et al., 2009; Ahmad et al., 2017; Junior et al., 2020). However, the delivery of therapeutically relevant amounts of  drugs exploiting the nasal route is strongly dependent on the availability of efficient nanoparticulate drug carriers (Mistry et al., 2015). The major approaches to increase drug bioavailability *via* intranasally administered nanoparticles are as follows: 1) the use of mucoadhesive polymers, increasing the local drug residence time; 2) incorporation of ingredients controlling the drug release rate; and 3) the employment of permeation enhancers (Sonvico et al., 2018).

Hybrid chitosan/lecithin nanoparticles display good mucoadhesion properties because of the cationic polysaccharide surface coating and good encapsulation properties for poorly soluble drugs due to the high lipid content (Gerelli et al., 2010; Şenyiğit et al., 2016). Together with a relatively small and narrow particle size distribution, these properties appear highly desirable for nanoscale-based formulations intended for nasal administration. However, in order to evaluate the suitability of lecithin/chitosan nanoparticles for the nasal delivery of SVT, *in vitro* drug release of nanoparticles was evaluated since it represents an important tool in the development of nanomedicines (Tinkle et al., 2014; Sainz et al., 2015). Conditions were selected so that they could be relevant for SVT release from nanoparticles in the physiological environment present in nasal cavities. In a relatively simple SNES, after an initial faster release rate evidenced for SVT-LCNs, the overall drug release from nanoparticles after 24 h did not differ from that of the control drug suspension. The decrease on the release rate of the nanoencapsulated SVT was attributed to the multilayered organization structure of nanoparticles hindering drug release at a later point. In fact, the polymer coating the nanoparticles acts as a drug-release barrier. Hence, the drug solubility and diffusion in/or across the polymeric or lipid layers become a determining factor in drug release. Moreover, the fact that SVT is a lipophilic drug which has a high affinity for the lipid component of the nanoparticles and its release could be hindered at an oil–water interface due to the low drug aqueous solubility has to be considered. Nevertheless, considering the specific conditions present in the nasal cavity and the rapid turnover of the mucus layer due to mucociliary clearance, the initial hours of *in vitro* drug release from the drug delivery system are critical to predict the drug bioavailability from a nasal formulation. Indeed, the nanoparticle formulation increased in 4-fold SVT release within the first 3 h compared to the drug suspension. However, considering the composition of the proposed nanocarrier, we hypothesized that enzymes naturally present in the nasal cavity trigger drug release toward hybrid lecithin/chitosan nanoparticles. In a different study, Barbieri et al. have shown the susceptibility of comparable lecithin/chitosan nanoparticles to biodegradation by gastrointestinal enzymes, promoting drug release and transport through the intestinal epithelium (Barbieri et al., 2015). Lysozyme and phospholipase A2 are antibacterial proteins naturally present in nasal secretions (Travis et al., 2001) and have the potential to degrade the two main components of the hybrid nanosystem. Lysozyme is capable to degrade chitosan (Nordtveit et al., 1996), while PLA2 catalyzes the hydrolysis of phospholipids, affecting the lipidic component of nanoparticles (Andresen et al., 2010). In drug release studies employing lysozyme, an increase in SVT release from nanoparticles was observed, with a doubling of the cumulative amount of the drug released compared to that of SNES (see Figure 2A). Interestingly, the addition of both enzymes determined a dramatic increase in the drug release rate with nearly all of the loaded SVT released within 7 h. SVT release from lecithin/chitosan nanoparticles could be actually triggered by the enzymatic action on nanoparticle structure biodegradation after deposition in the nasal cavity. These results corroborate the hypothesis that hybrid phospholipid/polysaccharide nanoparticles are mucoadhesive systems prone to biodegradation when they are in contact with the mucosal surface.

As aforementioned, the major drawback affecting the availability of nasally administered lipophilic drugs is the mucociliary clearance. Therefore, additional *in vitro* release studies were performed for SVT-LCNs dispersed in simulated artificial nasal mucus. A decrease in the cumulated amount of SVT released suggests an interaction between nanoparticles and mucins, resulting in the hampering of drug release and/or drug diffusion through the mucus network. In fact, the mucus layer has been recognized as a barrier for the diffusion of poorly water-soluble drugs, which interact with local glycoproteins and lipids, leading to an overall reduced diffusion rate (Sigurdsson et al., 2013). The presence of the antibacterial enzymes resulted in a boost of drug release in the presence of mucus, confirming once again the relevance of enzyme-triggered release of SVT from hybrid lecithin/chitosan nanoparticles in the nasal physiological environment. Thus, the proposed hybrid chitosan-coated nanoparticles interacted with the mucus secretion. Even though this interaction could prevent drug availability and consequent permeation through the nasal epithelium, their biodegradation by glycoside hydrolases and phospholipase enzymes naturally present in the nasal cavity could represent a new strategy to overcome the mucus barrier.

When considering these results, it is important to emphasize that drug release experiments in the present work employed Franz vertical diffusion cells. Even though *in vitro* experiments applying Franz cells have become one of the most frequent methods adopted for drug release studies of nonconventional drug dosage forms, they present the drawback of being a static diffusion method in which the dialysis membrane separating the donor and receptor compartments poses as a significant additional barrier to the diffusion of the drug (Ng et al., 2010; Modi and Anderson, 2013). All these considerations lead to the hypothesis that the released fractions of SVT could be even higher *in vivo*.

In the transport studies across the RPMI 2650 nasal epithelium model, the amount of SVT transported by nanoparticles was determined and compared with that obtained from a suspension of the free drug. Hybrid lecithin/chitosan nanoparticles increased the permeation of SVT by 11-fold. Interestingly, the amount of SVT found within the cells and the mucosa were similar for encapsulated SVT and free raw materials, suggesting that nanoparticles do not increase drug internalization and accumulation inside the epithelial nasal cells. The role of chitosan as a permeation enhancer is well known (Smith et al., 2004). However, it cannot be assumed that it is the only factor responsible for the hybrid nanoparticle permeation enhancement since in the case of SVT, one of the main limiting steps is the drug's poor water solubility. Considering that the release studies supported nanoparticle biodegradation, it is likely that LCN permeation enhancement was also supported by the biodegradation of the delivery system in the mucus barrier or by intracellularly providing a supplementary driving force to the drug diffusion across the mucosal barrier. In a recent study, Barbieri et al. investigated the permeation of tamoxifen across the intestinal epithelium of rats using lecithin/chitosan nanoparticles (Barbieri et al., 2015). Authors demonstrated that tamoxifen-loaded nanoparticles significantly increased the drug transport *ex vivo* across rat intestinal tissue when pancreatin or the lipase enzyme was present, suggesting that nanoparticle degradation increased the extent of drug intestinal permeation *via* an enhanced paracellular transport. Interestingly, when a semipermeable membrane was used to prevent nanoparticles contacting with the mucosal tissue, the amount of drug permeated from the nanoparticle formulation was similar to that obtained from the drug suspension. These results suggest a decisive role of the hybrid lecithin/chitosan nanoparticle structure in determining interactions at the biointerface and biodegradation processes within the mucosal tissue able to increase drug absorption and bioavailability.

Recent data pointed out that local brain immune activation has the capacity to facilitate and trigger the pathological and physiological modifications in neurodegenerative diseases. In particular, it has been shown that microglia cells play an important role in the management of inflammation in neurodegenerative pathologies. Beyond the lipid-lowering effect, many pleiotropic effects have been attributed to statins due to the inhibition of the mevalonic acid pathway and the formation of isoprenoid intermediates (Bellosta et al., 2000; Bedi et al., 2016). In fact, statins modulate inflammatory responses by 1) inhibiting signal transduction pathways activating pro-inflammatory transcription factors, such as nuclear factor κB; 2) by activating PPARα receptors and the anti-inflammatory activity; and 3) affecting the expression of the MHC II histocompatibility complex (Paumelle et al., 2006; McFarland et al., 2014), possibly through the disruption of cholesterol-containing microdomains, which transport and concentrate MHC-II molecules to the cell surface (Kuipers and Elsen, 2005; Kuipers et al., 2005).

In the present study, we have demonstrated an anti-inflammatory activity of both SVT-LCNs and SVT, with a greater effect exerted by the nanoformulation. In the case of SVT, our findings were in agreement with previously published data, indicating the capacity of statins to mitigate microglia and its activation and to reduce the production of neuroinflammatory mediators through the modulation of several signaling pathways (Bagheri et al., 2020). On the contrary, another previous study suggested a pro-inflammatory effect of statins able to increase the secretion of cytokines in Toll-like receptor–induced primary monkey microglia (Putten et al., 2012). The reason for this discrepancy among findings is not clear, but it may be very likely related to the use of different *in vitro* models and/or the type of pro-inflammatory stimuli.

Among the possible mechanisms to explain the enhanced anti-inflammatory activity of SVT-LCNs, we believe that there is a direct uptake and digestion of nanoparticles by cells, as suggested for other nanosystems (Tulbah et al., 2019). Indeed, it has been previously demonstrated that LCNs could effectively enter cells *via* the energy-dependent caveolae-mediated endocytosis and macropinocytosis (Chu et al., 2019). Moreover, surface charge has also been shown to be an important parameter determining the degree of particle uptake. For instance, Thiele et al. found that positively charged microparticles showed much greater uptake by human-derived macrophages and dendritic cells (Thiele et al., 2001).

In the present study, the enhanced anti-inflammatory activity detected for SVT-LCNs is to be attributed to the direct uptake and digestion of nanoparticles by the macrophages since empty nanoparticles did not present any effect in the release of IL-6 and TNF-α.

Notably, both SVT and STV-LCNs were more effective in suppressing IL-6 than TNF-α secretion. This differential effect may be very likely related to the extent of cytokine production triggered by LPS. Indeed, after LPS exposure, TNF-α reached the concentration of about 15,000 pg/ml, while IL-6 secretion was about 1,000× fold lower. This milder inflammatory setting may better highlight the inhibitory effect of statin formulation at lower concentrations. In addition, despite some concerns about potential cytotoxicity of cationic materials which have been raised by some studies in the past, LCN formulation was demonstrated to be well tolerated by human THP-1 glia-like cells. The association of nonconventional and pluripotent anti-inflammatory group of compounds such as statins with nanocarriers capable to deliver drugs directly from the nose to the brain constitutes an innovative and promising strategy in the treatment of neurodegenerative diseases.

## Conclusion

In summary, in the present work, we investigated the hybrid lecithin/chitosan nanoparticles designed as a platform for the nasal administration of SVT for treating neuroinflammatory disease. We observed that nanoparticle interaction with a simulated nasal mucus decreased nanoparticle drug release and/or slowed drug diffusion. We also evidenced the effect of enzymes present in nasal secretions, such as lysozyme and PLA2, in promoting drug release from the nanocarrier. Moreover, chitosan-coated nanoparticles enhanced SVT permeation across a human cell model of the nasal epithelium. Finally, we demonstrated an effect of SVT-LCNs in suppressing the pro-inflammatory stimulus in a myeloid cell line model for microglia. Taking the presented data into consideration, the encapsulation of SVT into lecithin/chitosan nanoparticles had been shown to be a beneficial strategy for the nasal delivery of lipophilic statins. Hybrid lecithin/chitosan nanoparticles not only provided a prompt drug release in biorelevant conditions, bypassing the mucus barrier and some enzymes naturally present in nasal secretions, but also enhanced the *in vitro* transport of SVT across a model of the nasal absorptive epithelium. The capability of exploiting innate immunity antibacterial enzymes to trigger drug release and possibly promoting drug permeation across cellular surfaces represents an innovative concept of controlled drug release for nanoparticle delivery carriers designed for nasal administration. In addition, the encapsulation of SVT was also demonstrated to improve the efficacy of the drug compound against a glial-like cell neuroinflammation model although we are aware that our “proof of principle” observation needs to be integrated with further evaluations on microglia cells with *in vivo* studies on relevant neuroinflammation animal models. Overall, it can be concluded that hybrid lecithin/chitosan nanoparticles appear as a highly attractive drug delivery platform for the nose-to-brain delivery of drugs with the potential to increase drug availability at the target organ and possibly its efficacy.

## Data Availability

The raw data supporting the conclusions of this article will be made available by the authors, without undue reservation.
