# Supplementary material for: Anti-Inflammatory Properties of Statin-Loaded Biodegradable Lecithin/Chitosan Nanoparticles: A Step Toward Nose-to-Brain Treatment of Neurodegenerative Diseases
Source: Front Pharmacol. 2021 Sep 24;12:716380. doi: 10.3389/fphar.2021.716380 (PMC8498028; doi:10.3389/fphar.2021.716380)
Supplement: Supplementary file 1 [file DataSheet1.DOCX]

Supplementary Material

# Supplementary Data

Supplementary Figure 1 reports the cellular viability of the THP-1 cells treated with increasing drug concentration (0.1 µM, 1µM and 10 µM) of simvastatin solution (SVT) or simvastatin-loaded nanoparticles (SVT-LCN) for 36 hours. Compared to the control group, neither the raw material nor nanoparticles formulation reduced the glia-like cells viability over all tested concentrations. Indeed, cells viability remained around 100% for all treated groups compared to untreated cells (no statistical differences recorded at 99% of confidence level).

The *in-vitro* efficacy of SVT-LCN was further assessed using again LPS-induced THP-1, as model of neuroinflammation. Prior to conducting efficacy studies, we preliminary evaluated time and dose-dependent effects of LPS on the stimulation of TNF-α and IL-6 release from THP-1 cells. Experimental results have shown that LPS have induced a significant pro-inflammatory response in the glia-like cell model at the concentration of 0.25 µg/ml causing a sustained release of IL-6 (Supplementary Figure 2, Panel A) and TNF-α (Supplementary Figure 2, Panel B) pro-inflammatory cytokines, achieving the maximum concentration after 6 hours after LPS stimulus.

## Supplementary Figures


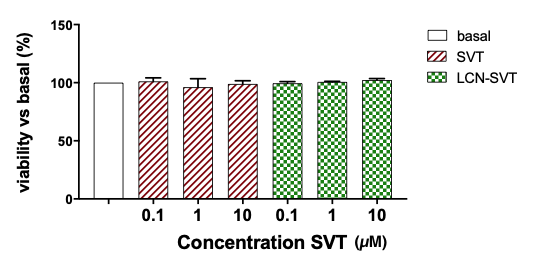


**Supplementary Figure 1.** MTT viability assay (%) of THP-1 human cell line treated up to 36 hours with increasing drug concentrations of simvastatin solution (SVT) and simvastatin-loaded nanoparticles (SVT-LCN). Data are expressed as mean percentage ± S.D. of cytokine secretion from at least three repeated measurements**.**

**Supplementary Figure 2.** **Panel A -** Secretion of IL-6 from THP-1 cells after 6 hours of LPS stimulus. **Panel B -** Secretion of TNF-α from THP-1 cells after 6 of LPS stimulus. Data are expressed as mean percentage ± S.D. of cytokine secretion from at least three repeated measurements.
